# From Cholera to Burns: A Role for Oral Rehydration Therapy

**DOI:** 10.3329/jhpn.v29i6.9902

**Published:** 2011-12

**Authors:** S.M. Milner, W.B. Green, M.E. Asuku, M. Feldman, R. Makam, D. Noppenberger, L.A. Price, M. Prosciak, I.N. van Loon

**Affiliations:** Johns Hopkins University School of Medicine,5505 Hopkins Bayview Circle, Baltimore, MD 21224, USA

**Keywords:** Burns, Cholera, Diarrhoea, Hydration, Oral rehydration therapy, Prospective studies, Rehydration, Shock, USA

## Abstract

According to the practice guidelines of the American Burn Association on burn shock resuscitation, intravenous (IV) fluid therapy is the standard of care for the replacement of fluid and electrolyte losses in burn injury of ≥20% of the total body surface area. However, in mass burn casualties, IV fluid resuscitation may be delayed or unavailable. Oral rehydration therapy (ORT), which has been shown to be highly effective in the treatment of dehydration in epidemics of cholera, could be an alternate way to replace fluid losses in burns. A prospective case series of three patients was carried out as an initial step to establish whether oral Ceralyte®90 could replace fluid losses requiring IV fluid therapy in thermal injury. The requirement of the continuing IV fluid therapy was reduced by an average of 58% in the first 24 hours after the injury (range 37-78%). ORT may be a feasible alternative to IV fluid therapy in the resuscitation of burns. It could also potentially save many lives in mass casualty situations or in resource-poor settings where IV fluid therapy is not immediately available. Further studies are needed to assess the efficacy of this treatment and to determine whether the present formulations of ORT for cholera need modification.

## INTRODUCTION

Oral rehydration therapy (ORT) was formulated for treating cholera in the 1960s (1,2). It was proven as a robust treatment in a mass casualty setting of cholera in refugees fleeing a war in East Pakistan (now Bangladesh) in 1971. In this setting with limited or no intravenous (IV) fluid therapy available, ORT alone decreased deaths from about 40% to 3% and was administered by friends and families of the cholera victims without any prior training (3). Later experiences in mass outbreaks of cholera in Peru and Rwanda confirmed its efficacy (4). Burn injuries often occur as mass casualty events. The initial loss of life is due to rapid loss of fluids and electrolytes into the areas affected by the burn. The current standard of care for burns over 20% of the total body surface area (TBSA) and 10% of the TBSA in children is prompt replacement of IV fluid using standardized formulae (5). In this case series, we reduced the volume of IV fluid replacement needed in three burned patients by starting treatment in the first 24 hours. We have reviewed the relevant literature and suggest that this may be an effective way to reduce the requirements of IV fluid therapy in burns. ORT has the potential of saving many lives in the event of mass thermal casualties or in resource-poor settings where transport, intensive care, and definitive surgical care may be delayed.

## MATERIALS AND METHODS

A prospective study of three patients with burn wounds (20-40% of TBSA) was performed. Fluid requirements were calculated according to the Parkland Formula [(Parkland Formula=4 cc/kg/% of TBSA, administered over 24 hours since the time of injury (50% given during the first eight hours and 50% given during the next 16 hours)] (5-7). For the first two hours, fluids were started at the Parkland goal with lactated Ringer's solution, minus 250 cc/hour. At the same time, Ceralyte®90 was started at 250 cc/hour via a Dobhoff tube. Fluids were then titrated at an interval of one hour as necessary by decreasing the IV fluids and increasing Ceralyte®90 (Table 1) to titrate the urine output to a target range of 0.5-1.0 cc/kg/hour. For urine outputs of <0.5 cc/kg/hour, ORT was increased by 100 cc/hour, and for urine outputs of >1 cc/kg/hour, IV fluids were decreased by 100 cc/hour. Gastric residuals were checked every two hours, and if greater than 300 cc, oral fluids were stopped.

**Table 1.** Composition of Ceralyte^®^90

Ingredient	Amount
Rice carbohydrate	40 g
Sodium	90 mEq/L
Potassium	20 mEq/L
Chloride	80 mEq/L
Citrate (base)	30 mEq/L
Osmolarity	270 mOsm/L

## RESULTS

All the patients tolerated the administration of ORT with gastric residuals of less than 300 cc which were monitored every two hours. The characteristics of the patients are defined in Table 2. We were able to replace a maximum of 77.5% of the IV fluid requirement, with a mean of 65.2% and range of 57.4-77.5% (Table 3). The patients maintained urine output within the target range throughout the course of the study, without electrolyte abnormalities, indicating adequacy of tissue perfusion with the ORT supplementation.

## DISCUSSION

We reviewed the literature on oral fluid replacement in burns but found only one report on the use of ORT in burns in humans, which treated burns (10-20%) in children. Resuscitation of IV fluid is the current standard of care for the treatment of patients with burns of ≥20% of the TBSA. Without intervention, the loss of fluids and electrolytes into burned tissues rapidly leads to hypotension and shock. In major burns, early resuscitation fluid is required to maintain circulation and adequate tissue perfusion. Volume losses occur most rapidly in the first 6-8 hours but continue for 18-36 hours or longer (8). Standardized formulae have been developed to guide the amount of IV fluids required for adequate resuscitation (9,10). However, mass thermal casualty events may disrupt logistics and limit access to resuscitation strictly by the IV route which requires skilled medical technical personnel and equipment.

ORT is a proven safe and effective alternative to IV resuscitation in epidemic cholera, a disease which causes rapid loss of fluids from the gut, leading to circulatory collapse and death. Oral rehydration solutions (ORS) take advantage of a robust back-up mechanism for intestinal absorption of salts and water, in addition to the normal absorption which is mediated by a sodium chloride-linked transport system at the brush border of the intestinal epithelium. This active transport moves sodium with chloride from the intestinal lumen into the cell. Cellular sodium potassium ATPase extrudes sodium into the lateral cell spaces, creating an osmotic and electrochemical gradient that produces a net flow of water and solutes from the lumen into the bloodstream (11,12).

**Table 2.** Patient demographics and 24-hour intravenous fluid requirements per Parkland Formula

Patient	Age (years)	% of TBSA burned	Weight (kg)	IVT requirement (Parkland Formula cc/24 hours)
1	48	20	60.5	4,840
2	44	25	104.4	10,450
3	31	40	111.8	17,888

IVT=Intravenous fluid therapy;

TBSA=Total body surface area

**Table 3.** Fraction and percentage of Parkland goal given by the enteral route

Patient	ORT/24 hours/Parkland goal	% given as enteral fluid in24 hours
1	2,613/4,840	60
2	8,098/1,0450	77.5
3	10,267/17,888	57.4

ORT=Oral rehydration therapy

This sodium-glucose transporter of the intestinal epithelium allows absorption of sufficient water and electrolytes to restore the large fluid losses even in severe diarrhoeal diseases when the normal absorptive mechanism is impaired (12). The World Health Organization (WHO) estimated that ORT currently saves more than three million lives every year from diarrhoeal diseases (13). The possibility that ORT could be an alternative to IV fluid therapy in replacing fluid losses due to burns has recently been reviewed (14).

In the present limited case series, we have shown that ORT decreased the amount of IV fluids needed to maintain adequate hydration in patients with severe burn wounds. By introducing a rice-based ORT—Ceralyte®90, there is the potential to substantially decrease the IV fluid requirement in the resuscitation of the thermally-injured patient, thereby decreasing the risk of such complications as line infection. Furthermore, the absorption offluid and electrolyte from the intestine is autoregulated by homeostatic mechanisms, including endocrine and mechano receptors. ORT is, therefore, less likely to result in fluid overload which can cause significant complications, such as compartment syndrome and pulmonary oedema, occasionally observed in burned patients receiving IV fluid therapy (15).

The use of ORT in burn resuscitation is currently being explored (16-18). In 1950, *JAMA* stated that “The use of oral saline is adopted as standard procedure in the treatment of shock due to burns, in the event of large scale civilian catastrophe” (19). However, we have found only one study comparing oral resuscitation with IV fluid therapy in small burns, although it has been demonstrated in animal models that oral resuscitation may be effective in meeting the fluid and electrolyte requirements following 40% of TBSA burns (20).

Paralytic ileus and reduced gastric emptying may limit oral resuscitation. The latter is described in critically-ill and burned patients. Gastric emptying may be reduced by opiates and sedation, and systemic inflammatory cytokines derived in thermal injury may also limit absorption (21). In our patients, we used a Dobhoff tube to facilitate administration of oral fluid. Vomiting did not occur, and gastric residuals did not impede the process. Results of studies suggest that vomiting may be reduced using hypotonic oral replacement solutions and buffering solutions containing citrate, lactate, or bicarbonate (13). However, vomiting is not considered an absolute contraindication to enteral resuscitation. Moreover, tube-feeding is routinely started soon after admission of burned patients and is thought to preserve nutrition, intestinal mucosal integrity, and facilitate healing (17,22).

The optimal composition of ORS for resuscitation in burn wounds has not yet been established. Kramer *et al.* have suggested a therapeutic advantage of the WHO oral rehydration fluid (14). In patients with cholera, Ceralyte®90, a rice-based ORS, has been proven superior to the WHO-ORS in increasing fluid absorption of the intestine and reducing diarrhoea (23). Rice starches provide glucose as substrate for the carrier-mediated co-transport mechanism without increasing luminal osmolality (24). Compared to fluid losses due to diarrhoea, fluid losses in burn wounds are difficult to measure but contain both extracellular fluid and potassium-rich cellular debris. During administration of Ceralyte to our patients, no electrolyte disturbances were, however, observed. To optimize the composition of ORT solutions for burn injuries, balance studies will be required, such as those that established the present highly-effective standard ORT solutions for fluid losses due to diarrhoea (25).

### Conclusions

The efficacy of ORT in diarrhoeal diseases is well-established. We have demonstrated a significant reduction in the 24-hour IV fluid requirement of patients with moderate burn injuries by a systematic supplementation with ORT using Ceralyte®90. We are convinced that the concept of this simple treatment holds promise in the future of fluid resuscitation of the thermally injured. Furthermore, in mass burn casualty situations where IV fluid therapy may be unavailable or delayed and in resource-poor settings, ORT could save many lives and deserves further investigation. The composition of ORS that would be optimal for burn resuscitation is equally worthy of further investigation.

## ACKNOWLEDGEMENTS

Conflict of interest: Dr. Greenough is a founding shareholder of Cera Products, Inc. which manufactures the rice-based ORS (Ceralyte®90) used in this study.
